# Supplementary material for: The Systems Biology Research Tool: evolvable open-source software
Source: BMC Syst Biol. 2008 Jun 29;2:55. doi: 10.1186/1752-0509-2-55 (PMC2446383; doi:10.1186/1752-0509-2-55)
Supplement: Additional file 1 — SBRT Archive. An archive of the current version of the Systems Biology Research Tool. [file 1752-0509-2-55-S1.zip › sbrt-1.4.0/doc/developers_guide/api/sbrt/kernel/math/algebra/proc/LinearSolverProcess.html]

LinearSolverProcess


|  |  |  |
| --- | --- | --- |
| **Overview** \| **Package** \| **Class** \| **Use** \| **Tree** \| **Deprecated** \| **Index** \| **Help** | |  |
| PREV CLASS   **NEXT CLASS** | **FRAMES**    **NO FRAMES**     **All Classes** |  |
| SUMMARY: NESTED \| FIELD \| CONSTR \| METHOD | DETAIL: FIELD \| CONSTR \| METHOD |  |


---


## sbrt.kernel.math.algebra.proc Class LinearSolverProcess<V>

```
java.lang.Object
  sbrt.kernel.math.algebra.proc.LinearSolverProcess<V>
```

**Type Parameters:**: `V` - the variable type.

**All Implemented Interfaces:**: KernelProcess<LinearSystemSolution<V>>

---

``` public class LinearSolverProcess<V> extends java.lang.Object implements KernelProcess<LinearSystemSolution<V>> ```

This class is used to solve systems of linear equations.

**Author:**
:   This class was written and documented by Jeremiah Wright while in the
    Wagner lab.

---

**Constructor Summary**

`LinearSolverProcess()`


| **Method Summary** | |
| --- | --- |
| `void` | `run(ResultsManager<LinearSystemSolution<V>> resultsManager)`             Solves the system of linear equations and adds the solution to the provided results manager. |
| `void` | `setEquations(java.util.Map<LinearComb<V>,java.lang.Double> equations)`             Sets the system of linear equations to be solver. |
| `void` | `setSolver(LinearSystemSolver<V> solver)`             Sets the linear system solver. |

**Methods inherited from class java.lang.Object**

`clone, equals, finalize, getClass, hashCode, notify, notifyAll, toString, wait, wait, wait`

**Constructor Detail**

### LinearSolverProcess

```
public LinearSolverProcess()
```


**Method Detail**

### setEquations

```
public void setEquations(java.util.Map<LinearComb<V>,java.lang.Double> equations)
```

:   Sets the system of linear equations to be solver.

    :   **Parameters:**: `equations` - linear combinations mapped to a double. Each key-value pair represents the left and right hand sides, respectively, of an equation.

---


### setSolver

```
public void setSolver(LinearSystemSolver<V> solver)
```

:   Sets the linear system solver.

    :   **Parameters:**: `solver` - the linear system solver.

---


### run

```
public void run(ResultsManager<LinearSystemSolution<V>> resultsManager)
```

:   Solves the system of linear equations and adds the solution
    to the provided results manager.

    :   **Specified by:**: `run` in interface `KernelProcess<LinearSystemSolution<V>>`
    :   **Parameters:**: `resultsManager` - the results manager to which the solution is added.


---


|  |  |  |
| --- | --- | --- |
| **Overview** \| **Package** \| **Class** \| **Use** \| **Tree** \| **Deprecated** \| **Index** \| **Help** | |  |
| PREV CLASS   **NEXT CLASS** | **FRAMES**    **NO FRAMES**     **All Classes** |  |
| SUMMARY: NESTED \| FIELD \| CONSTR \| METHOD | DETAIL: FIELD \| CONSTR \| METHOD |  |


---
